# Advancement flaps in perianal fistulizing Crohn’s disease: technical steps and current evidence

**DOI:** 10.1007/s10151-026-03325-5

**Published:** 2026-05-12

**Authors:** U. Aho Fält, P. Myrelid

**Affiliations:** 1 Surgery Research Unit, Department of Clinical Sciences Malmö, Lund University, Malmö, Sweden; 2 Department of Surgery and Gastroenterology, Skåne University Hospital, Malmö, Sweden; 3 Department of Biomedical and Clinical Sciences, Linköping University, Linköping, Sweden; 4 Clinical Department of Surgery in Linköping, Linköping, Region Östergötland Sweden

**Keywords:** Crohn’s disease, Anal fistula, Advancement flap, Surgery, Rectovaginal fistula

## Abstract

Fistulizing perianal Crohn’s disease is complex and negatively affects quality of life. Rectal advancement flap (AF) has been widely adopted as a sphincter-preserving procedure for treating complex anal fistulas. The European Crohn’s and Colitis Organisation (ECCO) guidelines on surgical treatment of Crohn’s disease state that AF is a therapeutic option for patients with Crohn’s disease and complex perianal fistulas. Despite its use in clinical practice, documented use of AF in treating fistulas related to Crohn’s disease remains limited. This narrative review aimed to examine the latest literature and discuss current evidence for the surgical treatment of fistulizing perianal Crohn’s disease using AF procedures. A PICO search was conducted in the electronic databases PubMed, Embase, and Google Scholar to identify studies published in English on the topic between 2018 and November 2025. In total, 11 studies were identified, including 305 patients with Crohn’s disease. Most patients received a draining loose seton prior to the AF procedure, while the proportion of patients on biological therapy or with a diverting stoma varied greatly. Reported success rates for clinical healing ranged from 23% to 91%, with median follow-up times reported between 7 months and 6 years (total range: 2 months to 10 years). Despite the inherent limitations of mostly retrospective and small studies, the available evidence supports the feasibility of AFs in fistulizing perianal Crohn’s disease, provided there is adequate fistula drainage and, importantly, optimized biological therapy to minimize luminal and perianal inflammation.

## Introduction

Perianal Crohn’s disease is a potentially severe form of Crohn’s disease that affects about 20% of patients. More than 10% of patients have perianal disease at or before their Crohn’s disease diagnosis and another 10% at time of their diagnosis [1, 2]. Anal fistulas and abscesses are reported as the most common lesions, occurring in 50% and 42% of cases, respectively, and being about 16 times more common in Crohn’s disease compared with the general population [1, 3]. The cumulative 20-year probability of developing a perianal fistula in Crohn’s disease is almost 30% [3]. Perianal fistulizing Crohn’s disease has a significantly greater impact on overall quality of life and several specific aspects compared with patients with Crohn’s disease alone [4].

Anal fistulas associated with Crohn’s disease are considered complex for several reasons. Patients have a higher risk of anal incontinence owing to diarrhea, secondary both to inflammation of the bowel and/or previous resections. Fistulas in Crohn’s disease may present with multiple tracts, secondary extensions, abscesses, and inflammatory cavities [5]. In female patients, anterior fistulas add further complexity because of a shorter anterior sphincter, proximity to the vagina, and the potential for pre-existing obstetric trauma.

Current evidence supports the hypothesis that multiple pathophysiological factors influence transmural inflammation and the development of persistent perianal Crohn’s fistulas. Microbiological factors, genetic predisposition, and epithelization of the fistula tract have been identified as contributors to perianal fistulation in Crohn’s disease. A persistent and dysregulated inflammatory response, characterized by elevated levels of matrix metalloproteinases (MMPs), proinflammatory cytokines, tumor necrosis factor (TNF), epithelial–mesenchymal transition (EMT), and abnormal distribution of luminal myofibroblasts, may impair wound healing. [6]

Rectal advancement flap (AF) has been widely adopted as a sphincter-preserving procedure for the treatment of complex anal fistulas. During the AF procedure, a flap consisting of the rectal/anal wall is mobilized to cover the excised and sutured internal orifice (IO) of the fistula. The thickness of the AF varies between studies and institutions, ranging from mucosal flaps to partial or full involvement of the internal anal sphincter (IAS). The external orifice (EO) and the peripheral fistula tract may be treated simultaneously with a core-out or by curettage. Recent European Society of Coloproctology (ESCP) guidelines for the treatment of cryptoglandular fistulas recommend AF for complex fistulas if ligation of the intersphincteric fistula tract (LIFT) and/or laser ablation are not feasible or have been unsuccessful [7]. The European Crohn’s and Colitis Organisation (ECCO) guidelines on surgical treatment of Crohn’s disease state that AF is a therapeutic option for patients with Crohn’s disease and complex perianal fistulas in the absence of active proctitis [8]. Despite frequent use in clinical practice, documented use of AF in the treatment of fistulas related to Crohn’s disease remains limited. The aim of this article is to review the latest literature and discuss current evidence for the surgical treatment of fistulizing perianal Crohn’s disease using the AF procedures.

### Methods

The review was conducted in accordance with the guidelines for the Scale for the Assessment of Narrative Review Articles (SANRA) and is presented as a narrative review. The analysis focused on literature addressing the use of AFs in fistulizing perianal Crohn’s disease, with emphasis on studies published after the most recent ECCO guidelines on surgical treatment of Crohn’s disease [8]. Articles reporting outcomes and technical aspects of AF, including those in combination with other treatments, were examined. Particular attention was given to reports on preoperative seton drainage, biological treatment, and diverting stomas.

Electronic databases PubMed, Embase, and Google Scholar were used to search for studies published between 2018 and November 2025. The searches were performed on 5 December 2025. Search terms included combinations of keywords and Medical Subject Headings (MeSH) “anal fistula,” “Crohn’s disease,” “advancement flap,” “rectal fistula,” and “Crohn disease” with different combinations of Boolean operators “AND” and “OR.” The search was limited to studies published in English. For Google Scholar, the screening was limited to the first ten pages filtered for the adequate time interval. The PICO criteria for inclusion are listed in Table 1. Case series and conference abstracts were excluded. Studies discussing only pouch-related fistulas were excluded.

**Table 1:** PICO criteria for study inclusion

PICO element	Keywords
P—Population	Patients with perianal fistulizing Crohn’s disease
I—Intervention	Advancement flap procedure (specified number of patients or procedures)
C—Comparison	No comparison group needed (observational studies accepted)
O—Outcomes	Clinical outcomes, such as success rate or recurrence rate

## Results

In total, 11 studies were included after the PICO search. The selected studies were published between 2018 and 2024 and examined patients who underwent surgery from 1995 to 2023. More detailed results are provided in Tables 2, 3, and 4. Four studies included patients with anal fistulas, and two studies reported results for ano/rectovaginal fistulas. Five studies included patients with both anal and ano/rectovaginal fistulas. Any pouch-related fistulas in the included studies were excluded from analysis.

**Table 2:** Included studies focused solely on anal fistulas after the PICO search

Title	Authors, year	Study design	Years	Patients	Flap type	Assessment	Follow-up	Follow-up time	Success rate	Failure rate	Other results
“Stem cell therapy in refractory perineal Crohn’s disease: long-term follow-up”	Wainstein et al. [12]	Prospective	2013–2016	8 pts with CD, 9 anal fistulas	RAF or MAF combined with adipose mesenchymal stem cells	CDAI, PDAI, MC, EUA, MRI, IBDQ	Clinical healing (complete or partial), CDAI, PDAIConfirmed	Median 31 months (21–37 months)	89% (8/9) fistulas with clinical healing; 1 fistula with partial healing	Not specified	Median PDAI significantly decreased
“Endorectal advancement flaps for perianal fistulae in Crohn’s disease: careful patient selection leads to optimal outcomes”	Roper et al. [15]	Retrospective, multicenter	2007–2017	39 pts with CD, 41 AFs	Wide-based, thick MAF	Colonoscopy, MR enterography, anoscopy or sigmoidoscopy	Clinical healing (complete closure of EO, no symptoms)	Average 27 months (1–256 months)	Primary healing 80% (33/41)	20% (8/41)	No recurrence in diverted pts; pts with longer IT before surgery more likely to achieve closure
“Ligation of the intersphincteric fistula tract and endorectal advancement flap or high perianal fistulas in Crohn’s disease: a retrospective cohort study”	Van Praag et al. [16]	Retrospective	2007–2018	37 pts with CD, 40 procedures; AF on 21 pts with CD	MAF with or without IAS fibers	MRI	Clinical healing (EO closure without discharge, pus, or feces), MRI, Vaizey IS	Median 15 months (5–44 months)	57% (12/21), 1 pt inconclusive, radiological healing 48% (10/21)	19% (4/21)	Significantly higher radiological healing rate when biological treatment and/or immunomodulatory used preoperatively, 43% pts with improved Vaizey IS
“Closing the internal opening with a rectal advancement flap increases the efficacy of mesenchymal stem cell injection for complex Crohn’s disease anal fistulas”	Fathallah et al. [17]	Prospective	2020–2023	68 pts with CD; AF on 20 pts with CD	Mesenchymal stem cells with or without AF	Anoperineal MRI, Magnifi-CD, MC, PDAI, JWS, CAF-QoL	Clinical remission (complete EO closure, no discharge or pain on pressure), MRI, Magnifi- CD, MC, PDAI, JWS, CAF-QoL	Median 16 months (9–25 months)	Complete clinical response 90% (18/20)	Not specified	Complete clinical response after suture 55% (*p* < 0.001), postop Magnifi-CD = 0 in 73% of AF pts (42% of suture pts, *p* = 0.093)

CD, Crohn’s disease; AF, advancement flap; RAF, rectal advancement flap; MAF, mucosal advancement flap; PAF, partial-thickness advancement flap; DAF, dermal advancement flap; IAS, internal anal sphincter; CDAI, Crohn’s Disease Activity Index; PDAI, Perianal Disease Activity Index; MC, Montreal classification; EUA, examination under anesthesia; MRI, magnetic resonance imaging; IBDQ, Inflammatory Bowel Disease Questionnaire; IS, incontinence score; IT, immunotherapy; JWS, Jorge–Wexner Score; CAF-QoL, Crohn’s Anal Fistula Quality of Life scale; EO, external orifice; pts, patients; Magnifi-CD, magnetic resonance novel index for fistula imaging in Crohn's disease; pts, patients

**Table 3:** Included studies focused solely on ano/rectovaginal fistulas after the PICO search

Title	Authors, year	Study design	Years	Patients	Flap type	Assessment	Follow-up	Follow-up time	Success rate	Failure rate	Other result
“What are the outcomes in patients referred to a tertiary referral center for Crohn’s rectovaginal fistula surgery?”	Sapci et al. [18]	Retrospective	2010–2020	20 pts with CD; AF on 7 pts with CD (RVF)	Semicircular AF	EUA with sigmoidoscopy	Clinical healing (no symptoms > 6 months and/or stoma closure)	Median 33 months (6–130 months)	85% (6/7)	Not specified	
“Surgical intervention is effective for the treatment of Crohn’s-related rectovaginal fistulas: experience from a tertiary inflammatory bowel disease practice”	Otero-Pinero et al. [14]	Retrospective	1995–2021	166 patients with CD; AF on 86 pts with CD (RVF)	RAF or TVAF	Not specified	Not specified	Median 6 years (1–10 years)	RAF 32% (22/68), TVAF 40% (6/15)	RAF 68% (46/68), TVAF 60% (9/15)	Overall success rate 72% (119/166) with multiple surgical interventions

CD, Crohn’s disease; AF, advancement flap; RVF, rectovaginal fistula; RAF, rectal advancement flap; TVAF, transvaginal advancement flap; EUA, examination under anesthesia; pts, patients

**Table 4:** Included studies focused on both anal and ano/rectovaginal fistulas after the PICO search

Title	Authors, year	Study design	Years	Patients	Flap type	Assessment	Follow-up	Follow-up time	Success rate	Failure rate	Other results
“Advancement flap procedure in Crohn and non-Crohn perineal fistulas: a simple surgical approach”	Bessi et al. [9]	Prospective	2006–2017	87 pts; 34 pts with CD (8 A/RVF)	PAF	MC, Cardiff-Hughes classification, HBI, PDAILE:	Clinical healing (no abscess, purulent discharge, EO, or further drainage)	Median 13 months (IQR 4–38 months)	68% (23/34) total, anal 73% (19/26), A/RVF 50% (4/8)	32% (11/34)	Absence of biological therapy in pts with CD associated with failure
“Sphincter-sparing surgery for complex anal fistulas: radiofrequency thermocoagulation of the tract is of no help”	Merlini I’Heritier et al. [11]	Case-matched	2006–2018	87 pts; AF on 11 pts with CD (5 AVFs out of total AFs)	Plicated IAS covered with MAF	Not specified	Clinical healing (no abscess, purulent discharge, EO, or further drainage)	Median 23 months (5–45 months) for all AF pts	64% (14/22) for all pts with CD (AF + TC)	32% (10/31) for all AFs, not specified for CD	AF and CD independent predictors for healing
“Long-term healing after complex anal fistula repair in patients with Crohn’s disease”	Mujukian et al. [13]	Retrospective	2008–2018	60 pts with CD; AF on 19 pts with CD (5 A/RVF)	14 MAF, 5 CAF	EUA or MRI	Clinical healing (EO closure, no discharge or pain)	Median 66 months (range 6–192 months)	23% total (MAF 14%, CAF 67%)	Not specified	Modest healing rates, LIFT predicts long-term healing
“Endorectal advancement flap with muscular plication in anovaginal and anterior perineal fistulas”	Egal et al. [10]	Retrospective	2011–2017	35 pts (27 AVF); 5 pts with CD	SubMAF with muscular plication	Not specified	Clinical healing (fistula closure, complete resolution of flatus or stool passage)	Median 31 months (4–72 months)	60% (3/5)	40% (2/5)	
“Healing of rectal advancement flaps for anal fistulas in patients with and without Crohn’s disease: a retrospective cohort analysis”	Seifarth et al. [19]	Retrospective (prospective database)	2010–2020	155 pts; AF on 55 pts with CD (16 RVF)	PAF	Not specified	Clinical (complete healing), EAUS	Median 7 months (2–12 months)	47% (26/55) total, anal 54% (21/39), RVF 34% (5/16)	53% (29/55)	No difference in healing rate between pts with or without CD

CD, Crohn’s disease; A/RVF, ano/rectovaginal fistula; AVF, anovaginal fistula; RVF, rectovaginal fistulas; AF, advancement flap; MAF, mucosal advancement flap; PAF, partial-thickness advancement flap; CVAF, cutaneous advancement flap; IAS, internal anal sphincter; PDAI, Perianal Disease Activity Index; MC, Montreal classification; EUA, examination under anesthesia; MRI, magnetic resonance imaging; HBI, Harvey–Bradshaw Index; IQR, interquartile range; LIFT, ligation of intersphincteric fistula tract; EO, external orifice; TC, thermocoagulation; pts, patients; EAUS, endoanal ultrasound

### Patient characteristics

A total of 305 patients with Crohn’s disease were included in the reviewed studies. At least 122 of these patients were reported to have ano/rectovaginal fistulas, but the exact proportion of patients with anal and ano/rectovaginal fistulas was not available in the reported inclusion data. Table 5 summarizes the results for the parameters that were the focus during the review process (Table 6).

**Table 5:** Results of the focus parameters from the included studies

Authors, year	Preoperative seton	Preoperative biological therapy	Ostomy	Related results
Wainstein et al. [12]^a^	100% (8/8) 4–6 weeks	50% (4/8) of CD	Not reported	
Bessi et al. [9]^a,b^	100% (87/87) of total pts with CD, median 6 months (4–11 months)	85% (29/34) of CD	Not reported	No preoperative biological therapy predictive of failure in pts with CD (*p* = 0.0122)
Merlini I’Heritier et al. [11]^a,b^	100% (62/62), unknown time	100% (11/11) of AF with CD	Not reported	
Roper et al. [15]^a^	76% (31/41) of total, duration not reported	32% (13/41) of total	20% (8/21) of total	Longer single-agent immunotherapy associated with better healing rate (*p* = 0.03)
van Praag et al. [16]^a^	95% (38/40) of total procedures, AF median 4 months (1–114)	43% (9/21) of AF (53% of LIFT)	24% (5/21) of AF (24% of LIFT)	Significantly higher radiological healing rate when biological treatment or immunomodulators used preoperatively
Mujukian et al. [13]^a,b^	68%, median 11 months (0–27 months)	38% (38/63) of total	32% (19/60) of total	Biologics or fecal diversion did not predict fistula healing in the total cohort
Egal et al. [10]^a,b^	100% (35/35) at least 6 weeks	Medical treatment optimized	Not reported	
Seifarth et al. [19]^a,b^	Not reported	65% (36/55) of CD	49% (27/55) CD (12% non-CD)	PTs with CD more likely to have a stoma (no effect on healing rate)
Otero-Pinero et al. [14]^b^	44% (73/166) of total	44% (73/166) of total	51% of total	Seton insertion had negative effect on healing rate, OR 0.42 (95% CI: 0.21–0.83)
Sapci et al. [18]^b^	Not reported	40% (8/20) of total	57% (4/7) of AF	Success rate 75% (6/8) for all pts on biological therapy, success rate 75% (6/8) for all pts with stoma
Fathallah et al. [17]^a^	Surgical EUA 2–3 weeks before to confirm good drainage	100% (20/20)	Not reported	

CD, Crohn’s disease; AF, advancement flap; EUA, examination under anesthesia; LIFT, ligation of intersphincteric fistula tract; OR, odds ratio; CI, confidence interval

^a^study with anal fistulas

^b^study with ano/rectovaginal fistulas

### Preoperative seton

Four studies reported that all patients were treated with preoperative loose seton [9–12]. Reported durations of seton drainage ranged from 4–6 weeks to a median of 6 months (range: 4–11 months). Other studies reported the preoperative use of seton drainage in 44–95% of cases [13–16], with reported durations varying from median 4 to 11 months (total range: 0–114 months). Some studies did not specify drainage duration [14, 15], and one study reported that a surgical examination under anesthesia was performed 2–3 weeks before AF operation to confirm good drainage [17]. Two studies did not mention the proportion of patients treated with a preoperative seton [18, 19].

### Biological therapy

The proportion of patients with Crohn’s disease on biological treatment before AF surgery was reported to vary between 50% and 100% [9, 11–13, 16, 19]. Three studies reported that the proportion of patients on biological treatment varied between 32% and 44%, with no specification regarding the patients with Crohn’s disease [14, 15, 18]. One study stated that the medical treatment of Crohn’s disease was optimized before surgery [10]. Bessi et al. reported a significantly higher healing rate of 96% (22/23) in patients who received biological treatment before surgery compared with 64% (7/11) in patients with failure, *p* = 0.0122 [9]. Roper et al. reported that patients with successful AF had a longer duration of immunomodulator therapy prior to AF compared with patients with failure, with average treatment times of 531 days and 262 days, respectively, *p* = 0.03 [15]. One study reported that anti-TNF treatment did not emerge as a significant predictor in univariate analysis [16] and another reported that biological therapy did not predict fistula healing in the total cohort [13]. But no data were given regarding whether these therapies were initiated owing to fistulizing disease or luminal activity in general, or active proctitis in particular. So far, the best data on the effect of advanced medical therapies are based on infliximab, but increasing data are emerging on the use of other biologicals as well as Janus kinase inhibitors and sphingosine-1-phosphate receptor modulators [8].

### Fecal diversion

There were major variations in the reported proportion of patients with fecal diversion in the included studies. The proportions ranged from 20% to 57% [13–16, 18, 19]. Five studies did not specify the proportion of patients with fecal diversion [9–12, 17]. Sometimes fecal diversion is mandated owing to rapidly progressive disease, which has been described by the TopCLASS consortium [20].

### Success rate and radiological healing

The reported success rates for clinical healing range from 23% to 91%, with median follow-up times reported between 7 months and 33 months (total range: 2–256 months) [9–13, 15–19]. One study reported a median follow-up time of 6 years (range 1–10 years) [14]. Seifarth et al. reported the use of endoanal ultrasound (EAUS) as part of the clinical follow-up [19]. Magnetic resonance imaging (MRI) was used for follow-up in two studies. In a Dutch report, van Praag et al. reported 60% (12/20) clinical healing for mucosal AF with or without IAS fibers in a study on high Crohn’s disease fistulas, while radiological healing was observed in 48% (10/20) of patients [16]. In a recent study on mesenchymal stem cells in combination with AF, Fathallah et al. reported a complete clinical response in 90% (18/20) of patients during a median follow-up of 16 months (range 9–25 months) [17]. In addition, 1 year postoperatively, 76% (52/68) of all included patients underwent MRI for follow-up. The Magnifi-CD score significantly decreased from 15.5 ± 4.7 at baseline to 5.9 ± 7.0 after 1 year (*p* < 0.001). Magnifi-CD score of 0 was achieved in 73% (8/11) of patients treated with AF in combination with mesenchymal stem cells.

## Discussion

### Preoperative seton

Modern management of perianal fistulizing Crohn’s disease is based on a combination of medical and surgical treatments. Active undrained infection is a contraindication to immunosuppressive therapy, such as anti-TNF agents. Therefore, a draining loose seton is usually placed to treat and prevent perianal sepsis in patients with anal fistulas related to Crohn’s disease. In the randomized controlled PISA trial, patients were assigned to treatment with chronic loose seton, anti-TNF treatment for 1 year, or surgery with either AF or LIFT after initiation of anti-TNF treatment, to compare medical and surgical management of perianal Crohn’s fistulas [21]. A significantly higher re-intervention rate in the medically untreated chronic seton group led to early termination of the study, and the authors concluded that seton treatment should not be recommended as the sole treatment for perianal fistulizing Crohn’s disease.

Most studies included in the review had a large proportion of patients who underwent drainage with seton before surgery. The duration of drainage varied widely, and longer drainage periods are sometimes needed in patients with Crohn’s disease to reduce inflammation and decrease the volume of inflammatory cavities. Extended drainage may also be necessary for biological treatment to take effect and help control luminal inflammation, particularly in the rectum. It has been discussed that longer drainage periods may negatively affect the fistula owing to the risk of epithelialization. In patients with Crohn’s disease, the possible negative effects of prolonged seton treatment may be outweighed by the positive effects of biological therapy, reduction of inflammatory cavities and anal stenosis, as well as general optimization of bowel function, since loose stool may increase the risk of mechanical flap failure. A negative effect of preoperative drainage was reported in a study of several different surgical procedures on patients with Crohn’s with rectovaginal fistulas [14]. Owing to the usually relatively short fistulas in the rectovaginal area, seton treatment may be less significant for treating inflammation and preventing sepsis, and the possible negative effects of seton treatment on fistula epithelialization may outweigh its benefits. In rectovaginal fistulas, the positive effects of adequate biological treatment, possibly combined with fecal diversion in severe cases, may be of great importance.

### Biological treatment

Current evidence and consensus support a combined medical and surgical approach to managing perianal fistulizing Crohn’s disease [8]. Many studies included in this review involve patients treated in the latter half of the 2000s, which likely contributes to the relatively low proportion of patients with Crohn’s disease receiving biological agents.

The PISA II trial included 94 patients with fistulizing Crohn’s disease who were randomized to either a surgical closure group (LIFT or AF) with short-term anti-TNF therapy or anti-TNF therapy alone or allowed to self-select their treatment arm [22]. Patients in the biological treatment arm had 6 weeks of seton drainage combined with biological monotherapy for 1 year. Patients in the surgical closure arm received approximately 8–12 weeks of seton drainage and 6–10 weeks of biological treatment before undergoing either LIFT or AF, at the surgeon’s discretion. The duration of anti-TNF therapy was about 4 months and was discontinued at the gastroenterologist’s discretion. No patients had diverting stomas prior to AF. The primary outcome was radiological healing on MRI at 18 months. Because the number of patients treated with LIFT or AF was not reported, the study was excluded in the PICO process. After a median follow-up of almost 6 years (IQR 5–7), radiological healing was observed in 27% (25/94) of patients, with significantly higher healing in 42% (15/36) of the surgical arm compared with 18% (10/55) in the biological treatment arm (*p* = 0.014). Long-term clinical closure occurred in 72% (26/36) of operated patients and 62% (34/55) of medically treated patients, with no statistically significant difference in closure rates (*p* = 0.18). Recurrences occurred in 22% (9/36) of patients in the surgical closure arm, but none of the 25 patients with radiological healing experienced recurrence. Therefore, treatment should aim for radiological healing as recurrences occurred even in patients with an almost completely fibrotic tract and only minimal activity on MRI. Anti-TNF therapy could be safely discontinued after surgical repair in patients with complete MRI closure and no other present indication for advanced medical therapy. The authors concluded that surgical closure should be considered in amenable patients with fistulizing perianal Crohn’s disease owing to favorable long-term outcomes.

### Technical notes

AF is often considered one of the established procedures in a fistula surgeon’s toolbox. In a recent international survey, nearly 4 out of 5 of the 492 surgeons, primarily from the USA and Europe, reported some experience with endorectal AF. Fewer than 15% of respondents performed more than ten procedures per year. Both partial (mucosa-submucosa) and full-thickness (including IAS) procedures were performed by slightly more than 50% of participants. [23] The AF procedure may be technically relatively easy to perform on a posterior fistula in an optimal cryptoglandular patient but can be extremely challenging in a stenotic anal canal of a suboptimally treated patient with Crohn’s disease. In our experience, fistula tissue and surgical planes are sometimes affected by inflammation long after mucosal healing, which makes both dissection of a tension-free flap and the preparation of the landing zone for the flap technically challenging. It is difficult to draw conclusions about flap thickness on the basis of the studies included in this review. Flap thickness may be of less importance in Crohn’s disease compared with cryptoglandular fistulas since healing of AF in the prior is considered to depend on a combination of precise eradication of the IO, effective closure of the mucosa, elimination of dead space, and optimal medical therapy for a sufficient period before and after surgery.

### Perianal fistula imaging

Recent ESCP guidelines for cryptoglandular fistulas recommend comprehensive preoperative imaging with MRI or EAUS [7]. Imaging techniques are important in the treatment of perianal fistulizing Crohn’s disease to define fistula type, assess the technical aspects of fistula anatomy and morphology, and quantify the amount of active inflammation. Both PISA II and the study on the combination of AF with mesenchymal stem cells by Fathallah et al. have used Magnifi-CD for preoperative assessment and follow-up after the procedure [17, 22, 24]. While many of the studies included in this review used clinical investigations for fistula classification, and some patient characteristics and treatment results were presented in terms of disease activity indexes, this highlights the need for pre- and postoperative imaging to ensure successful and optimal treatment of perianal fistulizing Crohn’s disease. It can be challenging to motivate patients without clinical symptoms to participate in postoperative MRI follow-ups owing to the inconvenient and time-consuming nature of the procedure. Technical developments in the latest generations of three-dimensional (3D) EAUS now allow acquisition of high-resolution images close to the anal canal, but challenges remain in assessing peripheral inflammatory masses. Further development of a validated EAUS-based CD activity score, as suggested by Pagano et al. in a recent review, would be valuable [25]. While MRI is crucial for research and treatment of advanced perianal Crohn’s disease, a more structured use of modern 3D EAUS by operating surgeons has significant potential as an effective bedside modality to complement MRI assessment.

Recent reports indicate that MRI measurements of fistula volume are associated with disease trajectory, with volume reductions correlating with classification shifts and clinical response [26]. From a surgeon’s point of view, this emphasizes the importance of timing the surgical procedure. After thorough imaging and mapping, combined with seton drainage and optimal medical treatment, inflammatory activity should be eliminated and dead space minimized before sacrificing tissue to create an AF. Injections of freshly collected autologous fat tissue have been described as a cost-effective and readily available alternative to mesenchymal stem cells. Reports on the use of this method for fistulizing perianal Crohn’s disease show promising results in clinical closure, radiological healing, and symptom improvement, even when fistulas persist or recur [27, 28]. Further studies are needed on the use of adipose tissue and other regenerative fistula treatments in combination with AF, as well as for symptom palliation in persisting fistulizing Crohn’s disease.

## Limitations

The retrospective design of most studies included in this review, along with substantial heterogeneity in patient populations, AFs, and fistula types, is the primary limitation of this review. Additionally, the often very small number of patients with Crohn’s included, or AFs performed, reduces the statistical strength of the conclusions drawn from these studies. A fundamental challenge of this review has been the wide range of surgical techniques classified as “advancement flap.” The included studies appear to cover a spectrum from mucosal buttonholes to extensive dissections of the lower rectal wall. Another challenge has been the heterogeneity of fistulas included in the reviewed studies, as anal fistulas and ano/rectovaginal fistulas are not clinically comparable. Moreover, differences in follow-up duration and the lack of standardized definitions of clinical and radiological healing further contribute to the heterogeneity. These diversities make it difficult to draw conclusions or generalize the results more broadly.

## Conclusions

This narrative review aimed to examine the latest literature and discuss current evidence for the surgical treatment of fistulizing perianal Crohn’s disease using the AF procedures. Despite inherent limitations, the review provides a synthesis of evidence published after the most recent ECCO guidelines. The available evidence supports the feasibility of AFs in fistulizing perianal Crohn’s disease, provided there is adequate drainage of the fistula and, importantly, optimal pre- and postoperative biological therapy to minimize inflammation. Concluding remarks are listed in Table 6. Future prospective, multicenter studies with standardized protocols and detailed descriptions of surgical techniques, potentially combined with modern individualized adjuncts, such as adipose tissue, are needed to strengthen the evidence. While it is important to evaluate treatment effects in terms of radiological healing, given the still modest reported success rates in treating this highly complex disease, more focus should also be placed on symptom control and effects on continence and quality of life, rather than solely on complete fistula healing.

**Table 6:** Concluding remarks on technical steps for advancement flap on perianal fistulizing Crohn’s disease

Step	Conclusions
Patient selection	On optimized medical treatment (biological therapy)
	No active proctitis, minimal luminal inflammation
	No or minimal anal canal stenosis (anal fistulas)
Timing	Fistula adequately drained with no signs of inflammation
	No major cephalic extensions, secondary tracts, or cavities of granulation tissue
	Wait for the medical treatment to take effect
Intraoperative remarks	Thorough dissection of a tension-free flap and preparation of a landing zone may be challenging—discontinue if significant fibrosis is present beneath the mucosa
	Precise eradication of the IO and proper closure of the fistula tract
	Minimize dead space
	Consider core-out of the EO and fistula tract up to the sphincter
Postoperative remarks	Plan the first follow-up in 3–4 months—if no signs of infection, re-evaluate clinical healing 6–12 months postoperatively
	Optimal medical treatment continued
	If complete clinical healing at 12 months, consider assessing radiological healing on MRI at 18 months before reducing medication

IO, internal orifice; EO, external orifice; MRI, magnetic resonance imaging

## Data Availability

No datasets were generated or analyzed during the review process.
